# Eco-Friendly Starch Composite Supramolecular Alginate–Ca^2+^ Hydrogel as Controlled-Release P Fertilizer with Low Responsiveness to Multiple Environmental Stimuli

**DOI:** 10.3390/gels9030204

**Published:** 2023-03-07

**Authors:** Supattra Tiamwong, Pratchayaporn Yukhajon, Pittayagorn Noisong, Maliwan Subsadsana, Sira Sansuk

**Affiliations:** 1 Materials Chemistry Research Center, Department of Chemistry and Center of Excellence for Innovation in Chemistry, Faculty of Science, Khon Kaen University, Khon Kaen 40002, Thailand; 2 Program of Chemistry, Faculty of Science and Technology, Nakhon Ratchasima Rajabhat University, Nakhon Ratchasima 30000, Thailand

**Keywords:** supramolecular hydrogel, phosphate, polysaccharide, starch, stimuli responsiveness

## Abstract

Environmentally friendly fertilizers (EFFs) have been developed to improve fertilizer efficiency and minimize adverse environmental impacts, but their release behavior under various environmental conditions has been less explored. Using phosphorus (P) in the form of phosphate as a model nutrient, we present a simple method for preparing EFFs based on incorporating the nutrient into polysaccharide supramolecular hydrogels using *Cassava* starch in the Ca^2+^-induced cross-link gelation of alginate. The optimal conditions for creating these starch-regulated phosphate hydrogel beads (s-PHBs) were determined, and their release characteristics were initially evaluated in deionized water and then under various environmental stimuli, including pH, temperature, ionic strength, and water hardness. We found that incorporating a starch composite in s-PHBs at pH = 5 resulted in a rough but rigid surface and improved their physical and thermal stability, compared with phosphate hydrogel beads without starch (PHBs), due to the dense hydrogen bonding-supramolecular networks. Additionally, the s-PHBs showed controlled phosphate-release kinetics, following a parabolic diffusion with reduced initial burst effects. Importantly, the developed s-PHBs exhibited a promising low responsiveness to environmental stimuli for phosphate release even under extreme conditions and when tested in rice field water samples, suggesting their potential as a universally effective option for large-scale agricultural activities and potential value for commercial production.

## 1. Introduction

As the growth of the global population has increased dramatically in the last few decades, together with an unstable world situation, food demand from the agricultural industry has surged accordingly [1]. As a result, fertilizers have been extensively utilized to attain higher productivity and quality. Specifically, rice farmers need large quantities of fertilizer every year. With the cost of fertilizer rising and the low global rice prices, farmers are less likely to cover their costs after each yearly harvest. Additionally, an over-application of fertilizer can negatively impact the environment or change the ecosystem, raising questions about the sustainability of modern agriculture [2,3]. Thus, improving utilization efficiency and preventing fertilizer loss to the environment are fundamental and likely meaningful goals of agricultural management.

Controlled-release systems have attracted more attention due to their release efficiency [4,5,6,7]. Various kinds of materials have been employed as carriers, such as inorganic materials [8,9,10], polymers [11,12,13,14], and composites [15,16,17]. These systems can reduce the loss of nutrients and improve efficiency to a certain extent. Furthermore, researchers have developed fertilizers with responsive properties to external stimuli such as pH, temperature, and light, that can control their release behavior [18,19,20,21]. However, there are still several disadvantages that can hinder large-scale production, such as difficult control of the release, possible risks of material carriers to the ecosystem, complicated fabrication processes, as well as high manufacturing costs.

Several strategies can be used to enhance fertilizer efficiency and mitigate the negative environmental impact. One such method is the utilization of environmentally friendly fertilizers (EFFs) [22,23]. EFFs can improve fertilizer efficiency by having a slow release rate of nutrients with a reduced initial burst. This means that the nutrients in the EFFs are released gradually over time, which allows plants to absorb them more efficiently and effectively. In contrast, commercial fertilizers often have a fast release rate of nutrients, which can result in an initial burst of nutrients that is too much for plants to absorb all at once. This can lead to nutrient loss through leaching and runoff, as well as reduced plant uptake and growth. By using EFFs with a slow release rate of nutrients, we can expect plants to use these nutrients more effectively, which can enhance fertilizer efficiency. Additionally, EFFs can also improve soil health and reduce environmental pollution, making them a more sustainable and eco-friendly option compared to traditional commercial fertilizers [22]. Natural materials commonly used in EFFs include chitosan [24,25], alginate [26], starch [27,28,29], cellulose [30,31], lignin [32], biochar [33,34], agricultural residues [35,36], and polydopamine [37]. As these materials degrade or convert into carbon dioxide, water, inorganic compounds, or microbial biomass, they are considered non-toxic to the environment, and offer various advantages over synthetic materials, such as low cost, excellent availability, eco-friendliness, and biodegradability. However, the negative characteristics of these materials limit their use in the preparation of efficient EFFs [22]. For instance, chitosan is typically soluble in acidic solutions, whereas cellulose is not soluble in most common solvents, including water and organic solvents [30,38]. Such solvent requirements can increase costs and have adverse effects on the environment. Sodium alginate easily degrades in the ionic environment of monovalent cations [39]. Moreover, a burst release of nutrients from starch-based EFFs is evident due to its low film-forming ability and high water dissolution [40]. Additionally, most EFFs do not respond directly to plant nutrient demands as they release nutrients quickly in the initial stage. Moreover, the release behavior of EFFs under various environmental conditions is not sufficiently understood.

This study presents a simple preparation of EFFs utilizing *Cassava* starch as a regulator and sodium alginate as a gelling agent with the coexisting incorporation of phosphate ions to form polysaccharide supramolecular hydrogel beads, namely starch-regulated phosphate hydrogel beads (s-PHBs). The s-PHBs were prepared using a straightforward approach in an aqueous medium at room temperature, with optimization of the amount of phosphate, alginate, and starch, as well as the pH of the solution. The obtained beads were initially evaluated regarding their physical appearance and further characterized with instrumental techniques. The swelling and degradation behaviors of the obtained beads were also studied. The phosphate release characteristics of the s-PHBs were examined in an aqueous solution and kinetically simulated with various mathematic models, which were compared with the phosphate hydrogel beads (PHBs). Importantly, their release behaviors were assessed under different environmental stimuli, including pH, temperature, ionic strength, and water hardness. Furthermore, the release efficiencies of these EFFs in surface water samples collected from rice fields located in various regions were investigated.

## 2. Materials and Methods

### 2.1. Chemicals and Reagents

All chemicals were used as received without further purification. Sodium alginate (C_6_H_9_NaO_7_) was purchased from Sigma-Aldrich (St. Louis, Missouri, USA). Tapioca starch ((C_6_H_10_O_5_)_n_) was obtained from R.S.Domestic Trading Co., Ltd. (Bangkok, Thailand). Potassium dihydrogen orthophosphate (KH_2_PO_4_) was acquired from BDH Prolabo (UK). Commercial phosphate fertilizer ((NH_4_)_2_HPO_4_) was purchased from a local market. Calcium chloride (CaCl_2_•2H_2_O) and sodium chloride (NaCl) were purchased from Ajax Finchem (Mumbai, India). Calcium carbonate (CaCO_3_) was obtained from Riedel-de Haën (North Carolina, USA). Deionized (DI) water, with a resistivity of 18.2 MΩ cm, was used in this study. A stock solution of 2.0 M KH_2_PO_4_ was prepared by dissolving the appropriate amount in 100 mL of DI water.

### 2.2. Preparation of s-PHBs

In a typical procedure for the preparation of s-PHBs, 2.0 g of sodium alginate was first dissolved in 50 mL of DI water. Then, 2.0 g of KH_2_PO_4_ as a source of PO_4_^3−^ ions and 2.0 g of starch were added slowly into the alginate solution. The mixture was continuously stirred at 500 rpm for 60 min to obtain a homogeneous suspension. After that, a 20 μL portion of the suspension was rapidly introduced into 100 mL of 0.1 M CaCl_2_ using a syringe with a needle diameter of 1.2 mm under magnetic stirring at 100 rpm for 30 min. The formed hydrogel beads were filtered and washed with DI water three times. Finally, these obtained beads were dried at room temperature under ambient conditions. Additionally, the PHBs were fabricated using a similar procedure, but without adding starch. The effects of amount of sodium alginate and starch, and the solution’s pH on the s-PHBs formation were studied.

### 2.3. Study of the Stability

The stability the of s-PHBs was evaluated with respect to their swelling and degradation behaviors. To study the swelling, the amount of absorbed water in the samples was examined after immersion in DI water at room temperature. In a typical procedure, weights of dry samples (*W*_d_) were measured before immersion. After that, the samples were immersed in water for a predetermined time. Then, weights of the wet samples (*W*_w_) were measured. The swelling behaviors of the s-PHBs were expressed as the percentage of absorbed water weights (%*W*_water_) in the samples obtained from the following equation:

%*W*_water_ = [(*W*_w_ − *W*_d_)/*W*_d_] × 100 (1)

Similarly, the degradation behaviors of s-PHBs were studied. After immersion, the samples were dried and weighed. The degradation (%D) of s-PHBs was determined as follows:

%D = [(*W*_d_ − *W*_i_)/*W*_d_] × 100 (2)

where *W*_d_ and *W*_i_ are the weights of the samples before and after the degradation study (immersion and drying), respectively. In comparison, the stability of PHBs was investigated. All stability tests of the samples were conducted in triplicate.

### 2.4. Study of Phosphate Release

The phosphate release from the s-PHBs, in comparison with the PHBs, was investigated in an aqueous solution at room temperature. In a typical procedure, 50 mg of fertilizer beads was soaked in 50 mL of DI water. The studied system was maintained at room temperature. Then, 1 mL of the supernatant was withdrawn at given time intervals, and 1 mL of DI water was added into the system to keep the solution volume constant. The release characteristics of the fabricated phosphate fertilizers were examined for a specific period of 48 h. The content of phosphate released from the fertilizers in the solution was quantified based on the phosphoantimonylmolybdenum blue complex reaction [41]. The release kinetics were expressed as the cumulative release ratio (%CRR) of phosphate in the following equation:

(3) $$\%\mathrm{CRR}=\left(\frac{C_{t}\cdot V_{total}+\sum_{0}^{t-1}C_{t}\cdot V_{t}}{m_{0}}\right)\times 100$$

where *C*_t_ and *V*_t_ are the concentration (mg/mL) and volume (1 mL) of the solution at time *t*, respectively. *V*_total_ is the total solution volume (50 mL) and *m*_0_ is the amount (mg) of phosphate in the obtained fertilizers. The experiments for studying the phosphate release of all samples were carried out in triplicate.

The responsive release of s-PHBs was further examined under various environmental conditions, including the solution’s pH, temperature, ionic strength (as NaCl concentration), and water hardness (as CaCO_3_ concentration). Additionally, the release behavior of the s-PHBs was evaluated in surface water samples collected during the rainy season from rice fields located in five different locations. All water samples were only filtered prior to use for studying the phosphate release.

To understand the release kinetics of both PHBs and s-PHBs, we employed four mathematical models, including the first-order kinetics, Higuchi, Ritger–Peppas, and parabolic diffusion, expressed as follows, respectively [42]:

ln(1 − *M_t_*/*M*_∞_) = −*kt* (4)

*M_t_*/*M*_∞_ = *kt*^1/2^ (5)

*M_t_*/*M*_∞_ = *kt^n^* (6)

(*M_t_*/*M*_∞_)/*t* = *kt*^−1/2^ + *b* (7)

where *M_t_/M_∞_* is the ratio of released phosphate at time *t*, *k* is the kinetic constant, *n* is the diffusion exponent, and *b* is a constant.

### 2.5. Characterization

The surface morphologies of the PHBs and s-PHBs were obtained using scanning electron microscopy (SEM, Mini-SEM, LEO, SNE-4500M). The functional groups of these samples were investigated by Fourier-transform infrared spectroscopy (FTIR, Bruker, TENSOR27). Thermal analysis of the samples was performed on a HITACHI STA7200 instrument at a heating rate of 10 °C/min from 30 °C to 800 °C under a nitrogen gas flow at 100 mL/min in open alumina pans with the use of α-Al_2_O_3_ as the standard reference material. UV–vis measurements were carried out using an Agilent Cary 60 UV–vis spectrophotometer.

## 3. Results and Discussion

### 3.1. Optimization for Preparing PHBs and s-PHBs

Alginate is generally composed of α-L-guluronate (G) and β-D-mannuronate (M) units, creating GM blocks. In the presence of Ca^2+^ ions, the -COO^−^ and –OH groups of the G blocks can form physically cross-linked gels, which can trap these cations in stable, continuous three-dimensional networks [43,44]. In this work, 0.1 M CaCl_2_ was used as a source of Ca^2+^ ions for cross-link gelation. For the PHBs, phosphate and alginate were first mixed thoroughly in an aqueous solution. For the s-PHBs, starch was added to the mentioned system. The ionic gelation reaction took place quickly at room temperature once drops of the mixture made contact with the Ca^2+^ solution. Two key parameters were considered, including the amount of sodium alginate and the pH of solution. In addition, an amount of starch was optimized for fabricating the s-PHBs.

First, the effect of the amount of sodium alginate on the formulation of PHBs was studied. The gelation was initially evaluated based on the physical appearance of the obtained gel beads. All beads were prepared in DI water using 2 g of KH_2_PO_4_ as a phosphate source, whereas sodium alginate was varied from 0.5 to 3.0 g. The results demonstrated that the PHBs with more stable spheres can be obtained using 2.0 g of sodium alginate, leading to an equivalent weight ratio of alginate to phosphate. Then, we considered the effect of the pH of solution for forming the PHBs using 2.0 g of alginate and 2.0 g of KH_2_PO_4_ at various pHs. It was observed that the PHBs were only partially formed under strongly acidic and alkaline conditions. At pH ≤ 4, carboxyl (–COOH) groups in alginate cannot be ionized and are electrically neutral, leading to a weakening of their interaction with Ca^2+^ ions and an inability to form hydrogel beads [45,46]. On the other hand, at pH ≥ 10, these –COOH groups are ionized and carry a negative charge, which enhances their interaction with Ca^2+^ ions. However, this increased ionization also causes the alginate polymer to absorb a significant amount of water and swell, which weakens the interaction between the –COOH groups and Ca^2+^ ions. As a result, the hydrogel may not form, or may form but be too weak due to excessive swelling. The results showed that PHBs with spherical shapes can be entirely formed in the solution pH range of 5–9. However, the PHBs prepared at pH = 5, denoted as PHBs-pH = 5, were seemingly more compact and rigid than other PHBs prepared at pH = 7 and 9.

**Figure 1:**
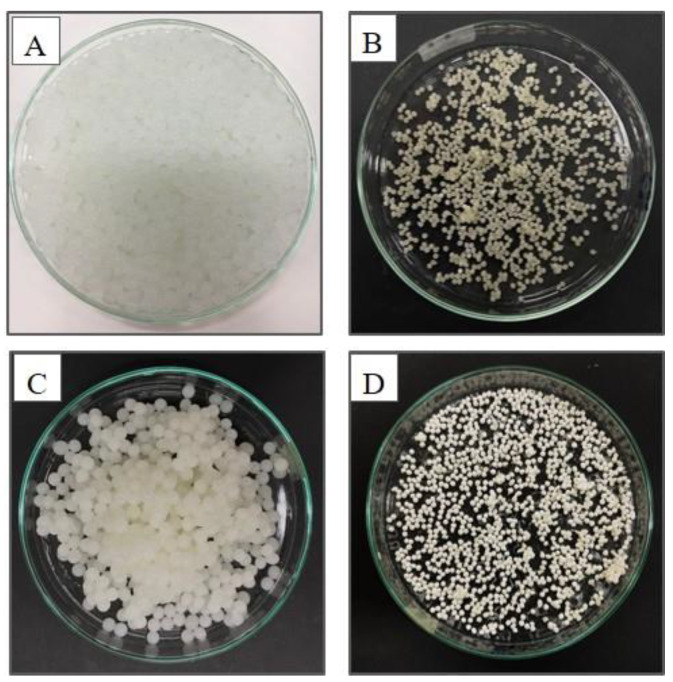
Photographs of wet and dry (**A**,**B**) PHBs and (**C**,**D**) s-PHBs, respectively.

Next, the s-PHBs were fabricated based on the same preparation conditions as the PHBs except for varying amounts of starch (0.5, 1.0, and 2.0 g). The results showed that white s-PHBs obtained with an optimal amount of 2 g of starch were more spherical and firmer than the others. Additionally, the effect of solution pH on the formation of s-PHBs was further evaluated. It was evident during the preparation that the suspension was clear at pH = 5, but cloudy at pH = 7 and 9, probably due to the dissolution of starch. Moreover, wet hydrogel spheres formed at pH = 5, denoted as s-PHBs-pH = 5, were smaller and their dried forms were more rigid. At pH = 5, the –COOH groups of alginate can be partially ionized and exist as carboxylate groups (–COO–), which can form hydrogen bonds with the –OH groups of starch and the phosphate groups (–PO_4_^3−^) [46]. The formation of hydrogen bonds between these components can lead to the creation of a dense supramolecular network in the hydrogel. Figure 1 shows photographs of wet and dry PHBs and s-PHBs prepared at pH = 5. The average diameter and weight were ~1.50 mm and 2.58 g for PHBs-pH = 5 and ~1.81 mm and 4.28 g for s-PHBs-pH = 5. In comparison, the PHBs and s-PHBs prepared in DI water, denoted as PHBs-DI and s-PHBs-DI, had an average diameter and weight of ~1.41 mm and 2.37 g for PHBs-DI and ~1.68 mm and 4.17 g for s-PHBs-DI. The greater size and weight of the s-PHBs were due to the presence of starch in the spheres. In conclusion, the optimal amounts of all ingredients for preparing the PHBs are 2 g of KH_2_PO_4_ and 2 g of alginate, whereas an additional 2 g of starch is required for preparing the s-PHBs. Although both PHBs and s-PHBs can be prepared in DI water and at pH = 5, their surface morphology and stability, as well as phosphate release characteristics, should be considered to acquire an optimal formulation of these EEFs with controlled release properties.

### 3.2. Surface Morphology of PHBs and s-PHBs

**Figure 2:**
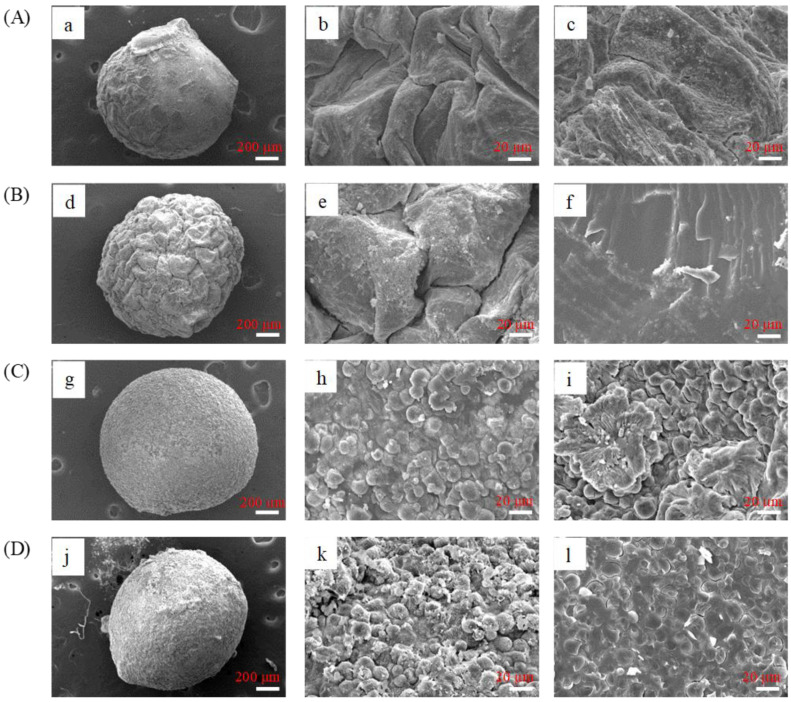
SEM images of (**A**) PHBs-DI, (**B**) PHBs-pH = 5, (**C**) s-PHBs-DI, and (**D**) s-PHBs-pH = 5 for whole bead (**a**,**d**,**g**,**j**), outer surface (**b**,**e**,**h**,**k**), and cross-section inner surface (**c**,**f**,**i**,**l**), respectively.

The surface morphology of the PHBs and s-PHBs was examined by SEM. Figure 2 shows SEM images of the whole, outer, and cross-sectional surfaces of PHBs-DI, PHBs-pH = 5, s-PHBs-DI, and s-PHBs-pH = 5. Figure 2A shows a smooth and compact external surface but a rough internal surface for PHBs-DI. It is expected that phosphate can be encapsulated into alginate networks during their formation. The alginate networks provide more active sites for interaction with phosphate ions and act as their protective shell to hinder their release into the solution. However, as seen in Figure 2B, a rough outer surface with residuals, but a smooth and compact inner surface, was observed in PHBs-pH = 5, possibly due to the effect of alginate solubility in acidic conditions. A significant difference in surface morphology for the s-PHBs is apparent, as shown in Figure 2C. The surface of the s-PHBs shows a higher degree of roughness than PHBs-pH = 5 because of the incorporation of starch molecules during gelation. The s-PHBs-pH = 5 in Figure 2D display the roughest surface with fairly large residuals, while their inner texture was relatively smooth and compact.

### 3.3. Chemical Structure of PHBs and s-PHBs

**Figure 3:**
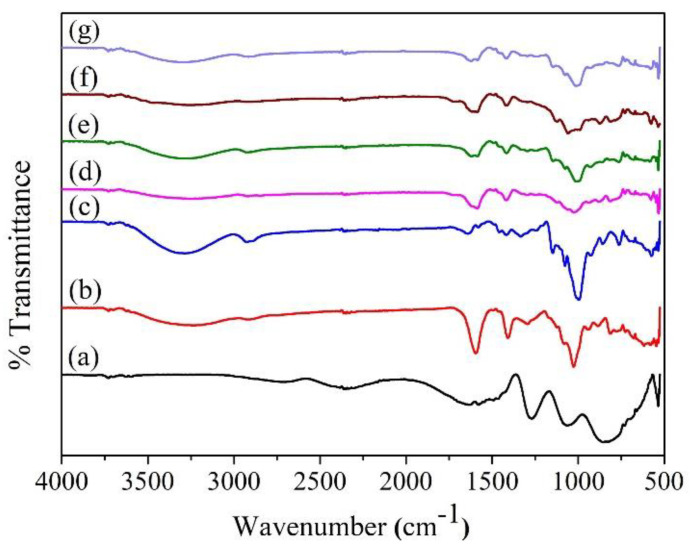
FTIR spectra of (**a**) KH_2_PO_4_, (**b**) alginate, (**c**) starch, (**d**) PHBs-DI, (**e**) PHBs-pH = 5, (**f**) s-PHBs-DI, and (**g**) s-PHBs-pH = 5.

FTIR measurements were conducted to determine the chemical interactions of all components in both the PHBs and s-PHBs. Figure 3 shows the FTIR spectra of KH_2_PO_4_, alginate, starch, and all fertilizer samples. As shown in Figure 3a, the three characteristic peaks at 662, 853, and 1064 cm^−1^ for KH_2_PO_4_ correspond to the polarization, symmetric stretching, and dipole vibration of PO_4_^3−^, respectively [47]. As shown in Figure 3b for alginate, the peaks at 1026, 1408, and 1595 cm^−1^ were assigned to the C-O-C stretching, COO^−^ asymmetric stretching of the carboxylate group, and COO^−^ symmetric stretching, respectively [48]. Additionally, a peak at 2924 cm^−1^ corresponds to the aliphatic group’s C-H stretching, and a broad peak at 3231 cm^−1^ is attributed to O-H stretching in the alginate molecule. For starch in Figure 3c, a characteristic peak was seen at 997 cm^−1^, resulting from the C-O-C skeletal vibration [42]. A peak at 1459 cm^−1^ corresponds to CH_2_ in-plane bending. A peak at 1639 cm^−1^ is ascribed to the H_2_O bending, indicating the moisture in starch. Moreover, a peak at 2927 cm^−1^ corresponds to the C-H stretching of an aliphatic group, and a broad peak was found at 3293 cm^−1^ and assigned to O-H stretching in the starch molecule. For PHBs in Figure 3d and PHBs-pH = 5 in Figure 3e, critical characteristic peaks of alginate were observed at 1059, 1417, 1588, 2915, and 3270 cm^−1^. The slight shift of peaks in the COO^−^ asymmetric stretching (carboxylate group) and COO^−^ symmetric stretching indicates electrostatic interaction with Ca^2+^ ions. Notably, the prominent characteristic peaks were found at 875 and 1123 cm^−1^, corresponding to the PO_4_^3−^ symmetric stretching and dipole vibrations. Compared with the spectrum of KH_2_PO_4_, these peaks shifted because of their interactions via hydrogen bonding with the carboxylate groups in alginate. These results indicate a successful incorporation of PO_4_^3−^ by crosslink gelation to form the PHBs. For the s-PHBs in Figure 3f and s-PHBs-pH = 5 in Figure 3g, characteristic peaks of PO_4_^3−^ at 661, 819, and 1076 cm^−1^, alginate at 1014, 1418, 1595, 2915, and 270 cm^−1^, and starch at 933, 1455, 2925, and 3293 cm^−1^ were observed. These results confirmed the complete incorporation of all components in the formation of s-PHBs. The apparent shifts or new peaks imply that the hydrogen bonds are fundamental interactions between phosphate groups, carboxylate groups of alginate, and hydroxyl groups of starch composite in the s-PHBs [46]. In addition, it was observed that the FTIR spectrum of both PHBs and s-PHBs prepared in DI water and solution pH = 5 were identical. The results imply no influence of pH on the chemical structure of these phosphate fertilizers.

### 3.4. Stability of PHBs and s-PHBs

**Figure 4:**
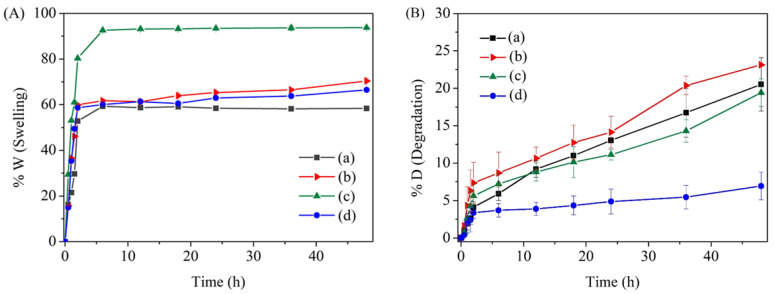
Plots for (**A**) swelling and (**B**) degradation behavior of (**a**) PHBs-DI, (**b**) PHBs-pH = 5, (**c**) s-PHBs-DI, and (**d**) s-PHBs-pH = 5.

The stability of fertilizers is considered an essential factor for their practical use in various environments. Additionally, the stability of fabricated fertilizers can relate to their phosphate release kinetics in an aqueous solution [49,50]. For this reason, the physical and thermal stabilities of all PHBs and s-PHBs were evaluated. First, the swelling of PHBs and s-PHBs was studied. Figure 4A shows the swelling behavior of PHBs-DI, PHBs-pH = 5, s-PHBs-DI, and s-PHBs-pH = 5 in solution. The results indicated a rapid increase in swelling of all samples in the first 6 h. After that, the swelling reached equilibrium at about 60% for PHBs-DI, PHBs-pH = 5, and s-PHBs-pH = 5, whereas s-PHBs-DI had over 90% swelling. If the immersion time was extended up to 48 h, the swelling of PHBs-pH = 5 and s-PHBs-pH = 5 gradually increased to 70.4% and 66.5%, respectively. However, the swelling of PHBs-DI and s-PHBs-DI was almost constant at 58.4% and 93.7%, respectively. The results imply that adding starch to EFFs significantly increases their swelling, especially those prepared in DI water. However, this effect can be avoided by preparing these EFFs in acidic conditions at pH = 5.

Next, the degradation of both PHBs and s-PHBs in an aqueous solution was studied. Figure 4B shows the degradation behavior of PHBs-DI, PHBs-pH = 5, s-PHBs-DI, and s-PHBs-pH = 5. It was found that the degradation of all samples surged rapidly in the first 2 h due to the dissolution of components on the external surface and a rapid release of phosphate. After that, the degradation increased to different degrees when the immersion time was prolonged to 48 h. The degradation of PHBs-DI, PHBs-pH = 5, and s-PHBs-DI increased progressively up to 20.5%, 23.1%, and 19.4%, respectively. However, the s-PHBs-pH = 5 degraded slightly up to 6.9%. This second stage of fertilizer degradation may be associated with their phosphate release. Additionally, a slight reduction in the pH of solution was observed, possibly caused by the acidic degradation products from gel beads [51]. The results suggest a slow degradation rate of s-PHBs-pH = 5, compared with the other beads, demonstrating that s-PHBs-pH = 5 are more stable in solution. This finding possibly results from the gelation reaction occurring in acidic conditions with the presence of starch, which can act as a structural regulator during their formation.

**Figure 5:**
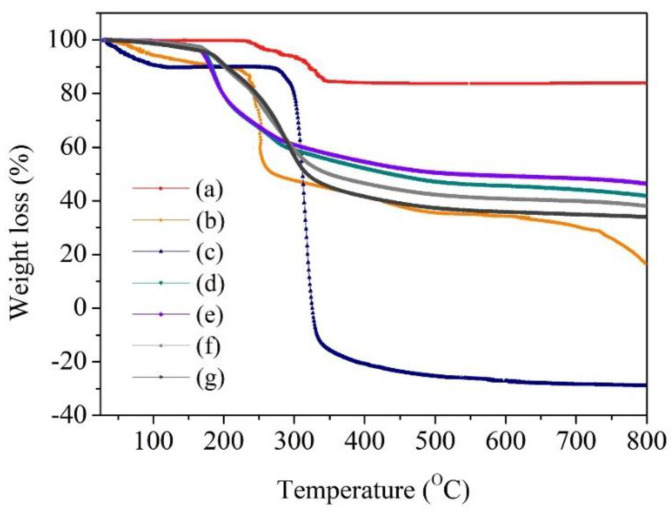
TGA curves of (**a**) KH_2_PO_4_, (**b**) alginate, (**c**) starch, (**d**) PHBs-DI, (**e**) PHBs-pH = 5, (**f**) s-PHBs-DI, and (**g**) s-PHBs-pH = 5.

In addition, thermal analysis of all samples was conducted to evaluate their stability and verify their components. Figure 5 shows the TGA curves of KH_2_PO_4_, alginate, starch, PHBs-DI, PHBs-pH = 5, s-PHBs-DI, and s-PHBs-pH = 5. It was observed that KH_2_PO_4_ powder undergoes a two-step weight loss. The first step involves dehydration, and the second step at 300 °C results from the decomposition of phosphate [16]. The s-PHBs exhibited higher stability compared to the other beads, indicating that the network structure of starch provides rigidity to the s-PHBs. However, s-PHBs-pH = 5 was more thermally stable than s-PHBs-DI. This finding suggests that the acidic condition induces a strong ionic environment, resulting in the formation of rigid beads. For s-PHBs-pH = 5, two stages of weight loss were observed. The initial weight loss from 25 to 200 °C was attributed to water dehydration. The second stage, from 200 to 800 °C, possibly corresponds to the decomposition of water, KH_2_PO_4_, alginate, and starch [16,52,53]. As a result, better phosphate release can be expected for s-PHBs-pH = 5 under the influence of temperature.

### 3.5. Release Behavior of PHBs and s-PHBs

**Figure 6:**
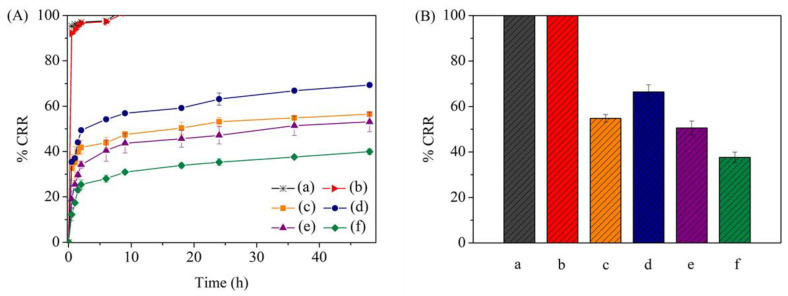
(**A**) Cumulative release ratio of (**a**) KH_2_PO_4_ powder_,_ (**b**) fertilizer 18-46-0, (**c**) PHBs-DI, (**d**) PHBs-pH = 5, (**e**) s-PHBs-DI, and (**f**) s-PHBs-pH = 5 in aqueous solution and (**B**) their plots of average phosphate release in the last 24 h.

The phosphate release kinetics of both PHBs and s-PHBs were examined over 48 h in DI water at room temperature (~26.0 °C), and compared with KH_2_PO_4_ powder and commercially available 18-46-0 fertilizer. Figure 6A shows the phosphate release profiles of all samples over time. It was evident that the release rate of KH_2_PO_4_ powder and commercial fertilizer was fast due to their high dissolubility. The phosphate release from both samples surged to over 90% within 6 h, and the complete release was achieved after 12 h. In contrast, sustained release was observed for PHBs and s-PHBs, indicating that alginate and starch helped to retain the phosphate release. However, the pH of the solution used to prepare these fertilizers plays an important role in their release characteristics. Among the PHBs, PHBs-pH = 5 had a slower release rate than PHBs-DI. Conversely, s-PHBs-DI had the fastest release rate, compared to PHBs-DI and PHBs-pH = 5. This finding is consistent with the swelling and degradation characteristics of s-PHBs-DI, as the starch composite in s-PHBs-DI undergoes swelling, thus accelerating the release of phosphate. A significant improvement in the sustained release was achieved in s-PHBs-pH = 5. To highlight this, an average value of the %CRR of all samples in the last 24 h of release is reported in Figure 6B. The phosphate release order of all samples is as follows: KH_2_PO_4_ powder >18-46-0 fertilizer > s-PHBs-DI > PHBs-DI > PHBs-pH = 5 > s-PHBs-pH = 5. These results suggest that the solution pH = 5 is most appropriate for fabricating phosphate-incorporated polysaccharide supramolecular hydrogels. In addition, using starch under this pH condition in sample preparation diminishes the effect of burst release, considering initial release rate within the first 3 h. This reduction is probably due to the immobilization of PO_4_^3−^ ions in the supramolecular networks of the alginate and starch composites. Moreover, there is a possibility that the phosphorylation of starch occurs during the preparation at pH = 5 [54]. This phosphorylated starch can help regulate the phosphate release because the addition of phosphate groups to starch can make it more resistant to the environment, slowing down the release rate and making the release more controlled. Consequently, s-PHBs-pH = 5 exhibited a slow release rate of phosphate, with a reduced initial burst, which can potentially enhance the fertilizer efficiency.

**Figure 7:**
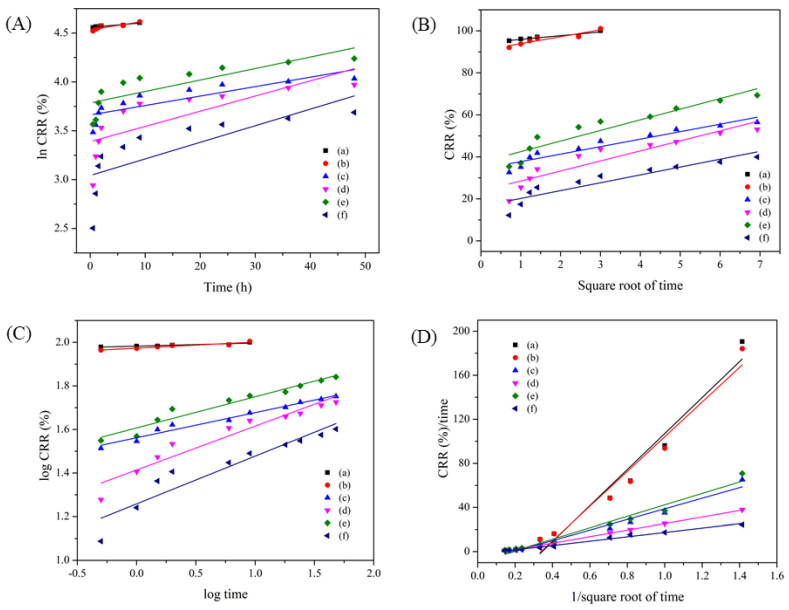
Linear plots of kinetic models for phosphate release of (**a**) KH_2_PO_4_, (**b**) DAP, (**c**) PHBs-DI, (**d**) PHBs-pH = 5, (**e**) s-PHBs-DI, and (**f**) s-PHBs-pH = 5: (**A**) first-order, (**B**) Higuchi, (**C**) Ritger–Peppas, and (**D**) parabolic diffusion.

In addition, four mathematical models including first-order, Higuchi, Ritger-Peppas, and parabolic diffusion, were used to understand the release mechanism of phosphate from the developed EFFs. From Equations (4)–(7) in Section 2.4, the linear plots of each model for the phosphate release profiles of all samples are shown in Figure 7, and the corresponding fitting parameters are summarized in Table 1. Based on the correlation efficiency (R^2^) of each mathematic model, the KH_2_PO_4_ powder and 18-46-0 fertilizer followed the first-order equation with fast release rates. The phosphate release of these samples mainly depends on their dissolution in solution [22]. Both PHBs-DI and PHBs-pH = 5 followed the parabolic diffusion model, indicating controlled diffusion from external surfaces [16,42]. The *k* values were 48.02 for PHBs-DI and 29.56 for PHBs-pH = 5. These values were lower than those of KH_2_PO_4_ powder and 18-46-0 fertilizer, indicating the success of sustained release by incorporating PO_4_^3−^ into alginate gel beads. It was noted that the *k* value of PHBs-pH = 5 was lower than that of PHBs-DI, suggesting the effect of solution pH during the synthesis on their release rates. For the s-PHBs, the release behaviors fitted well with the parabolic diffusion model, similar to the PHBs. The *k* value of s-PHBs-DI was 51.8, whereas it was 19.75 for s-PHBs-pH = 5. A high *k* value of s-PHBs-DI can be associated with their swelling. These results confirmed that s-PHBs-pH = 5 has better controlled release in solution than s-PHBs-DI.

### 3.6. Release Responsiveness of PHBs and s-PHBs to Environmental Conditions

In this section, the prepared PHBs-pH = 5 and s-PHBs-pH = 5 are abbreviated as “PHBs” and “s-PHBs”, respectively. Their responsive release behavior was examined under various environmental conditions, including pH, temperature, ionic strength, and water hardness, as described below.

#### 3.6.1. Effect of pH

**Figure 8:**
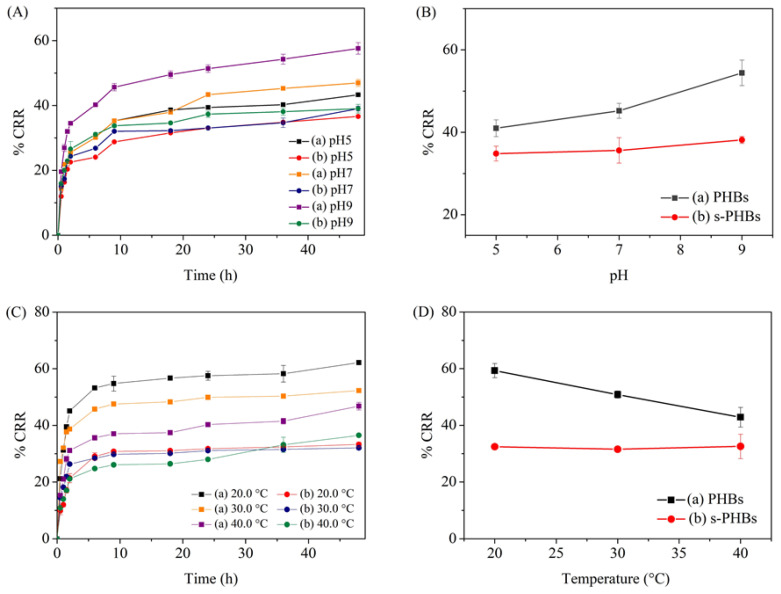
Effect of pH on phosphate release; (**A**) the cumulative release ratio and (**B**) their corresponding plots for phosphate release in the last 24 h of (**a**) PHBs and (**b**) s-PHBs at different pHs. Effect of temperature on phosphate release; (**C**) the cumulative release ratio and (**D**) their corresponding plots for average phosphate release in the last 24 h of (**a**) PHBs and (**b**) s-PHBs at different temperatures.

The effect of pH on phosphate release by the PHBs and s-PHBs was investigated at different pHs. Figure 8A shows the release profiles of PHBs and s-PHBs over 48 h at pH = 5.00, 7.06, and 9.08. The results indicated a more substantial influence of the solution pH on the phosphate release from PHBs than for s-PHBs. As shown in Figure 8B, the phosphate release of PHBs increased when the pH of the solution increased. It was nearly a 50% increase in phosphate release when the pH solution was increased from pH = 5 to 9, possibly due to its structural change. In an alkaline solution, the spheres of the PHBs dissolved absolutely, leading to an increased phosphate release into the solution. This result confirmed a responsive release property of the PHBs towards the pH of the solution. In contrast, the influence of the pH on the phosphate release characteristics of the s-PHBs was minimal. This result specifies that the controlled-release behavior of the s-PHBs can be realized by incorporating PO_4_^3−^ ions into the supramolecular networks of starch during the gelation process. Based on the four kinetic models, the results showed that nearly two fitting equations, including Ritger–Peppas and parabolic diffusion, can be applied for the phosphate release of the PHBs and s-PHBs. This result exhibited that it is not a simple sustained release process [42]. If considering the R^2^ values, however, the release better matched the parabolic diffusion equation (R^2^ > 0.9914 for PHBs and R^2^ > 0.9888 for s-PHBs), emphasizing that diffusion was the primary release process. However, the dissolution or ion exchange processes can be taken into account. It was also found that the *k* values of the PHBs were almost equivalent at pH = 5 (*k* = 23.0927) and pH = 7 (*k* = 23.0152) but higher at pH = 9 (*k* = 30.7679). This fluctuation shows a strong effect of the alkaline conditions on their release characteristics. For the s-PHBs, an insignificant change in the *k* values in the studied pH ranges was apparent (*k* = 19.8885 for pH = 5, *k* = 22.7501 for pH = 7, and *k* = 24.3620 for pH = 9). This finding indicates a reduced effect of solution pH on the release kinetics of the s-PHBs, confirming their low release responsiveness.

#### 3.6.2. Effect of Temperature

The effect of the solution temperature on the phosphate release of PHBs and s-PHBs was studied at 20.0, 30.0, and 40.0 °C. Figure 8C shows the release profiles of PHBs and s-PHBs at different temperatures. The results specify a strong effect of temperature on the phosphate release of the PHBs. The phosphate release was reduced when the solution temperature was increased, as shown in Figure 8D, indicating that the PHBs are temperature-responsive. Interestingly, a lesser effect of the solution temperature on the phosphate release of the s-PHBs was observed. This discovery can result from the supramolecular networks that help reduce the degradation of alginate gel spheres and prevent the release of PO_4_^3−^ ions into the solution by solid attraction. This result is supported by the TGA results in Figure 5, showing improved thermal stability of the s-PHBs. According to the kinetic models and considering the *R*^2^ values, the release behaviors of both the PHBs and s-PHBs still followed parabolic diffusion at all studied solution temperatures [16,42]. However, more deviation of the *k* values under the influence of the solution temperature was noticeable in the PHBs (*k* = 24.4656–41.1478 for PHBs and *k* = 14.9384–22.3779 for s-PHBs). The results highlight the advantage of using starch in the preparation of gel beads. The thermal tolerance of the s-PHBs makes them more suitable for practical applications in any season as their phosphate release efficiency remains unchanged.

#### 3.6.3. Effect of Ionic Strength

**Figure 9:**
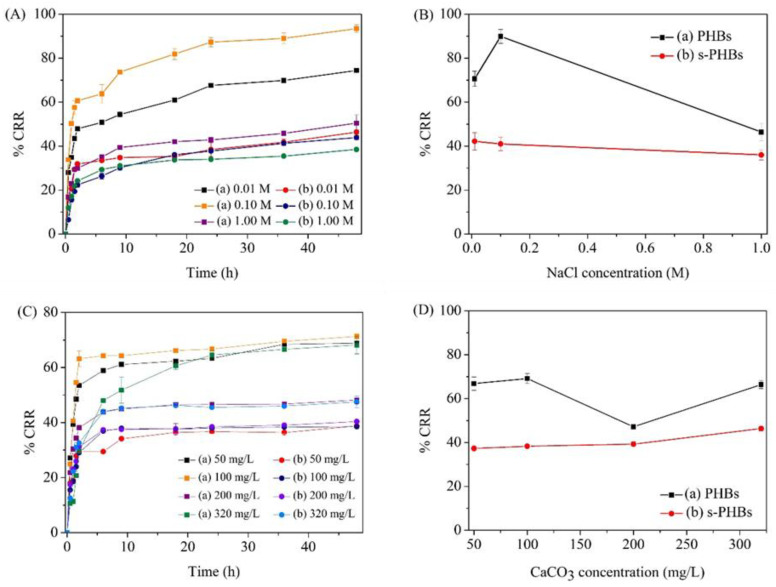
Effect of ionic strength on phosphate release; (**A**) the cumulative release ratio and (**B**) their corresponding plots for average phosphate release in the last 24 h of (**a**) PHBs and (**b**) s-PHBs at different NaCl concentrations. Effect of water hardness on phosphate release; (**C**) the cumulative release ratio and (**D**) their corresponding plots for average phosphate release in the last 24 h of (**a**) PHBs and (**b**) s-PHBs at different CaCO_3_ concentrations.

As a large number of ions are present in groundwater, it is impossible for applied fertilizers to avoid interactions with these ions via adsorption, ion exchange, and other effects. For this reason, the effect of ionic strength on the release behavior of both PHBs and s-PHBs was studied. In this study, NaCl salt was adopted to simulate the ionic environment. The phosphate releases of PHBs and s-PHBs were evaluated at three NaCl concentration levels (0.01, 0.10, and 1.00 M) over 48 h at room temperature. Figure 9A,B shows the release profiles of PHBs and s-PHBs and their %CRR plots for phosphate release in the last 24 h at different NaCl concentrations, respectively. The results highlight a significant effect of the ionic environment on the release of phosphate from PHBs. At 0.10 M NaCl, %CRR increased to over 90%, compared to 70% at 0.01 M and 50% at a non-ionic atmosphere (no NaCl added). The increased phosphate release of the PHBs at higher NaCl concentrations may be due to the strong influence of Na^+^ and Cl^-^ ions on the surface of the PHBs. This interaction can cause a structural change of gel spheres, leading to a high release rate. Nevertheless, the phosphate release of the PHBs unexpectedly decreased at 1.00 M NaCl. An excess of Na^+^ and Cl^-^ ions can restrain ion exchange with the PHBs, thus affecting their outer texture. This phenomenon can lead to a turn-off mode that suppresses the release of PO_4_^3−^ ions from the PHBs. This result links the responsive release property of the PHBs to changes in ionic strength. In contrast, the s-PHBs exhibited a trivial reduction in phosphate release in response to an ionic environment. The %CRR only slightly decreased at a high NaCl concentration (1.00 M), perhaps because of the supramolecular network structure of starch in the s-PHBs that can diminish the adsorption or ion exchange of Na^+^ and Cl^-^ ions, even at excessive levels of Na^+^ and Cl^-^ ions. In addition, the calculation results showed that the release kinetics of PHBs relate better to parabolic diffusion (*R*^2^ = 0.9913–0.9948) at all NaCl concentrations with a high variation in the *k* values (*k* = 26.5449–54.7095). Similarly, the releases of s-PHBs followed a parabolic diffusion process (*R*^2^ = 0.9885–0.9932). However, less fluctuation of the *k* values was obtained (*k* = 12.5229–25.9063). This minor responsive release property of the s-PHBs to ionic strength can make them suitable for practical uses in any ionic environment.

#### 3.6.4. Effect of Water Hardness

The effect of water hardness on the phosphate releases of both PHBs and s-PHBs was examined. In this study, the water hardness was expressed as the concentration levels of CaCO_3_. The release efficiency of the samples was evaluated at four CaCO_3_ concentrations (50, 100, 200, and 320 mg/L), representing different degrees of water hardness. Figure 9C shows the kinetic release behavior of the PHBs and s-PHBs at room temperature under different levels of water hardness over 48 h. The experimental results displayed a strong effect of water hardness on the phosphate release of PHBs. As shown in Figure 9D, a notable deviation of the release was found in the PHBs, suggesting their responsive property to water hardness. This consequence is probably due to the strong interaction of Ca^2+^ and CO_3_^2−^ ions in the solution with PHBs that can disturb the release of phosphate to some degree. Significantly, the phosphate release of PHBs was in a turned-off mode at 200 mg/L of CaCO_3_, possibly because of an overkill of CO_3_^2−^ ions that can suppress the diffusion of PO_4_^3−^ ions from the PHBs into the solution. In the case of the s-PHBs, however, the effect of water hardness on their release was minimal up to 200 mg/L of CaCO_3_. These results suggest that the s-PHBs tolerate the governing cationic and anionic environment. At this CaCO_3_ level, the release of s-PHBs was not disturbed by other ions. However, numerous Ca^2+^ and CO_3_^2−^ ions at 320 mg/L of CaCO_3_ can interact with the outer surface of s-PHBs, leading to a high degree of degradation. Thus, the diffusion of PO_4_^3−^ ions into the solution increased. From the calculation models, the results indicated that the releases of both PHBs and s-PHBs had a better fit with the parabolic diffusion equation at all studied CaCO_3_ concentrations (*R*^2^ = 0.9053–0.9962 for PHBs and *R*^2^ = 0.9399–0.9967 for s-PHBs). If considering the *k* values, a substantial decrease was found in PHBs from *k* = 44.1718 at 50 mg/L of CaCO_3_ to *k* = 14.8381 at 320 mg/L of CaCO_3_, whereas less deviation was seen in s-PHBs (*k* = 21.9177–27.1645). These results emphasize the advantage of starch for better controlled-release characteristics under the influence of water hardness. To conclude, the responsive property of the s-PHBs towards the water hardness was low. Thus, s-PHBs can be considered for many applications, where their release efficiency remains relatively constant under all environmental conditions.

### 3.7. Applicability of s-PHBs in Agricultural Water Samples

**Figure 10:**
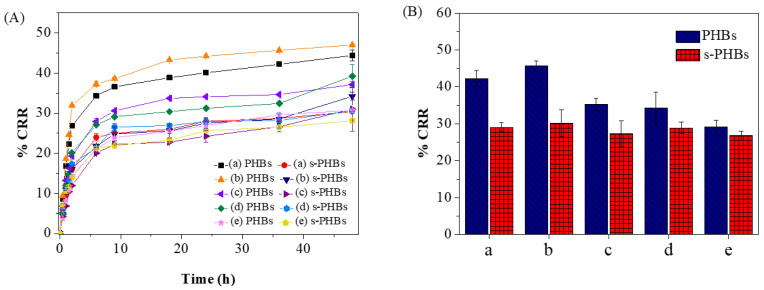
(**A**) Cumulative release ratio of PHBs and s-PHBs and (**B**) their plots for phosphate release in the last 24 h in rice field water samples located in different provinces; (**a**) Khon Kaen, (**b**) Nakhon Ratchasima, (**c**) Buriram, (**d**) Maha Sara Kham, and (**e**) Surin.

The applicability of both PHBs and s-PHBs was tested in agricultural water samples. Surface water samples were collected during the planting season from the rice fields in five provinces, including Khon Kaen, Nakhon Ratchasima, Buriram, Maha Sara Kham, and Surin, Thailand. As shown in Figure 10, the phosphate release of the PHBs fluctuated according to the quality of the water samples. In contrast, the release efficiency exhibited by s-PHBs remained consistent across all water samples with minimal deviation. These results demonstrate the potential practical use of s-PHBs in agriculture through their consistent release efficiency, indicating their cost-effectiveness and sustainability. The addition of starch to EFFs has the potential to reduce fertilizer costs in various ways. Firstly, starch is a cheap and plentiful natural polysaccharide, making it an attractive substitute for more costly synthetic polymers or other additives [49]. Secondly, the use of starch in EFFs can enhance their physical and chemical stability, resulting in reduced losses and increased efficacy, which can ultimately decrease the required amount of fertilizer to attain the desired crop yield. Finally, the regulated release features of starch-containing EFFs can promote more efficient nutrient absorption by plants, reducing the overall amount of fertilizer required and ultimately lowering the cost of fertilizer application. Overall, the inclusion of starch in EFFs presents an economical and sustainable solution for agricultural fertilization. As efficient EFFs, the developed s-PHBs are biodegradable, eco-friendly, and versatile. Additionally, the straightforward fabrication process of s-PHBs, which did not require any organic solvents or energy, makes them well-suited for large-scale commercial production.

## 4. Conclusions

In summary, environmentally friendly fertilizers (EFFs) with low responsiveness of nutrient release to environmental conditions were successfully produced by incorporating a nutrient (in this case, phosphorus in the form of phosphate) into hydrogels made from Cassava starch and alginate through Ca^2+^ cross-linking gelation at pH = 5 and room temperature. The addition of polysaccharide supramolecules influenced the texture, degradation, and swelling of the hydrogel EFFs, improving their stability. A controlled release of the nutrient was observed, with a reduced initial burst release rate. Under various environmental conditions, the prepared EFFs showed limited fluctuations in their release efficiency, demonstrating their low responsiveness to environmental stimuli. This behavior could be attributed to the strong hydrogen bonding interactions between the supramolecular networks and the nutrient. Therefore, these nutrient-incorporated polysaccharide supramolecular EFFs with sustained release in response to environmental conditions could have broad applicability in agriculture and are suitable for large-scale production.

## Figures and Tables

**Table 1:** Kinetic fitting parameters of each mathematical model for the phosphate release of all samples.

Model Parameter	First-Order		Higuchi		Ritger–Peppas			Parabolic Diffusion		
	*k*	*R^2^*	*k*	*R^2^*	*k*	*R^2^*	*n*	*k*	*R^2^*	*b*
KH_2_PO_4_	0.0078	0.9852	2.8615	0.7868	95.9867	0.7001	0.0222	163.1695	0.9607	−56.0595
18-46-0 fertilizer	0.0087	0.9844	3.2899	0.8895	94.0052	0.9122	0.0283	157.5069	0.9642	−53.3066
PHBs-DI	0.0097	0.7297	3.5643	0.9199	36.4738	0.9741	0.1153	48.0215	0.9801	−9.2635
PHBs-pH = 5	0.0155	0.5923	4.7828	0.8612	25.9484	0.9283	0.2012	29.5638	0.9974	−4.1631
s-PHBs-DI	0.0117	0.6828	5.0542	0.8950	40.4531	0.9535	0.1432	51.7961	0.9721	−9.4696
s-PHBs-pH = 5	0.0170	0.5727	3.7395	0.8620	18.1305	0.8955	0.2193	19.7474	0.9889	−2.3884

## Data Availability

Not applicable.
